# Brain MRI and EEG overemployment in patients with vasovagal syncope: results from a tertiary syncope unit

**DOI:** 10.1186/s12872-023-03615-y

**Published:** 2023-11-21

**Authors:** Masih Tajdini, Amirmohammad Khalaji, Amir Hossein Behnoush, Hamed Tavolinejad, Arash Jalali, Saeed Sadeghian, Ali Vasheghani-Farahani, Somayeh Yadangi, Farzad Masoudkabir, Ali Bozorgi

**Affiliations:** 1 Cardiovascular Diseases Research Institute, Tehran Heart Center, Tehran University of Medical Sciences, Tehran, Iran; 2 Cardiac Primary Prevention Research Center, Cardiovascular Diseases Research Institute, Tehran University of Medical Sciences, Tehran, Iran; 3 School of Medicine, Tehran University of Medical Sciences, Poursina St., Keshavarz Blvd, Tehran, 1416634793 Iran; 4 Department of Epidemiology and Biostatistics, School of Public Health, Tehran University of Medical Sciences, Tehran, Iran

**Keywords:** Vasovagal syncope, Neurologic examination, Magnetic resonance imaging, Electroencephalography, Diagnosis

## Abstract

**Background:**

The diagnosis of vasovagal syncope (VVS) is mainly based on history-taking and physical examination. However, brain Magnetic Resonance Imaging (MRI) and Electroencephalogram (EEG) are commonly used in the diagnostic course of VVS, despite not being indicated in the guidelines. This study aims to find the possible associated factors with the administration of brain MRI and EEG in patients with VVS.

**Methods:**

Patients with a diagnosis of VVS from 2017 to 2022 were included. Several demographic and syncope features were recorded. The association of these was assessed with undergoing MRI, EEG, and either MRI or EEG. Univariate and multivariable logistic regression models were also used to calculate odds ratios (OR) and 95% confidence intervals (CI).

**Results:**

A total of 1882 patients with VVS were analyzed, among which 810 underwent MRI (43.04%), 985 underwent EEG (52.34%), and 1166 underwent MRI or EEG (61.96%). Head trauma (OR 1.38, 95% CI 1.06 to 1.80), previous neurologist visit (OR 6.28, 95% CI 4.24 to 9.64), and gaze disturbance during syncope (OR 1.75, 95% CI 1.13 to 2.78) were all positively associated to the performance of brain MRI/EEG. Similar results were found for urinary incontinence (OR 2.415, 95% CI 1.494 to 4.055), amnesia (OR 1.421, 95% CI 1.053 to 1.930), headache after syncope (OR 1.321, 95% CI 1.046 to 1.672), and tonic-clonic movements in head-up tilt table test (OR 1.501, 95% CI 1.087 to 2.093). However, male sex (OR 0.655, 95% CI 0.535 to 0.800) and chest pain before syncope (OR 0.628, 95% CI 0.459 to 0.860) had significant negative associations with performing brain MRI/EEG.

**Conclusion:**

Based on our findings, performing MRI or EEG was common among VVS patients while it is not indicated in the majority of cases. This should be taken into consideration to prevent inappropriate MRI/EEG when there is a typical history compatible with VVS.

## Introduction

Syncope, characterized by transient loss of consciousness (TLOC), and in particular vasovagal syncope (VVS), is one of the most common causes of hospital visits in the United States and all over the world, with an incidence of 8.6 per 1000 people per year [1]. Its pathophysiology includes a decrease in peripheral vasculature resistance sometimes combined with bradycardia [2, 3]. The cornerstone of VVS diagnosis is proper history taking; however, electrocardiogram (ECG) and supine blood pressure measurement, as suggested by the guidelines, are routinely used [3]. Several additional measures have also been utilized, including the head-up tilt test (HUTT) [4, 5], electrophysiological study [6], echocardiography [7], exercise stress training [8], and in rare cases, coronary angiography [3].

In the absence of focal neurologic findings or neurological features suggesting seizure, neurologic evaluation including brain magnetic resonance imaging (MRI) and electroencephalogram (EEG) is not indicated in patients presenting with syncope [2, 3]. The European Society of Cardiology (ESC) guidelines for syncope recommends avoiding brain MRI in uncomplicated syncope and suggests it should be used in case of any sign of Parkinsonism, ataxia, or cognitive impairment [3]. However, a significant percentage of patients with syncope have already undergone MRI and/or EEG when visiting the tertiary centers. Based on American Heart Association (AHA) guidelines [2], brain MRI was used in 11% of 397 patients and led to syncope diagnosis in 0.24%. In addition, EEG was used in 52% of 2084 patients leading to a diagnosis in 0.7% [2].

Although studies have been conducted to investigate the diagnostic value of neurological examination in patients with syncope, none of them evaluated patients with only VVS. Moreover, the predictors of this overuse have not been investigated so far. In this study, we investigated the prevalence of MRI and EEG usage before presentation to the syncope unit of Tehran Heart Center [9] in addition to baseline variables’ association with this misuse.

## Methods

### Design

This registry-based prospective study was held at the syncope department of Tehran Heart Center, Tehran University of Medical Sciences, Tehran, Iran. This department is a referral clinic dedicated to the management of patients with syncope, especially VVS and situational types [10]. Patients diagnosed with VVS referred to this center from 2017 to 2022 were enrolled. The ethics committee of Tehran University of Medical Sciences approved this study (IR.TUMS.MEDICINE.REC.1401.716), and the study was performed according to the Declaration of Helsinki.

### Population

All patients that were diagnosed with VVS based on history, either from the patients, families, or bystanders, in addition to relevant physical examination and Calgary Syncope Symptom Score (CSSS) ≥ -2 [11] were included. In case of uncertain causes, VVS was diagnosed after the exclusion of other causes and clinical judgment. Noteworthy, although all patients underwent HUTT, the diagnosis was not only based on HUTT results. Patients with evidence of postural orthostatic tachycardia (≥ 30 bpm increase in heart rate after a 5-minute stand test) or orthostatic hypotension (≥ 20/10 mmHg decrease in blood pressure [BP] after a 5‐minute stand test) were excluded. Moreover, we excluded patients with valvular heart disease, a decrease in BP more than 50 mmHg after carotid sinus massage in patients older than 40 years with uncertain diagnosis, ventricular pause > 3s, and left ventricular ejection fraction ≤ 40% in transthoracic echocardiography. Patients with a cerebrovascular accident (CVA) history, those with carotid Doppler sonography showing stenosis (hemodynamically relevant stenosis > 50%) before presentation to the syncope clinic, and patients with other neurological examination indications such as seizure suspicion were also excluded.

### Variables and their measurement

Baseline variables were assessed, including demographics (age, sex, weight, height, and body mass index), past medical history of diseases (including diabetes, hypertension, dyslipidemia, valvular heart disease, chronic kidney disease, arrhythmia, and history of myocardial infarction, all based on patients’ self-report), syncope features (number of spells, symptoms in each spell, bystander observations, trauma, location of syncope, and initiating factors), HUTT features (symptoms during the test, orthostatic blood pressure, and type of response), and family history (seizure, syncope, and sudden cardiac death). Movements during syncope and HUTT were recorded and were defined as tonic or clonic movements and myoclonus. Autonomic symptoms included palpitations, diaphoresis, heat feeling, flushing, or abdominal discomfort. Previous brain MRI and EEG assessments were also investigated. Patients were also categorized based on brain MRI and EEG assessment, in addition to any of these two (brain MRI or EEG assessment).

### Statistical analysis

Mean and standard deviation (SD) were used to describe normally distributed variables. Three comparisons were made based on: (1) previous brain MRI assessment, (2) previous EEG assessment, and (3) previous brain MRI or EEG assessments. For the comparison of categorical variables, Pearson chi-squared statistical test was applied, while the independent sample t-test was used for continuous variables. Univariate and multivariable logistic regression models based on all variables relevant based on clinical judgment were also applied for all three neurological assessments using all features mentioned to calculate the odds ratios (OR) and 95% confidence intervals (CI). The area under the receiver operating characteristic curve (AUC) is an evaluation metric to calculate the performance of any classification models including logistic regression. We used the AUC metric to evaluate the discriminatory power of the multivariable logistic regression model in discriminating patients with or without previous brain MRI or EEG. A *P* of < 0.05 was considered statistically significant. All analyses were performed using R version 4.0.4 (R Core Team [2020]. R: A language and environment for statistical computing. R Foundation for Statistical Computing, Vienna, Austria).

## Results

### Population

Among all the patients who were diagnosed with VVS, 48 patients had a history of CVA, and 261 patients had abnormal carotid Doppler ultrasound which was excluded from the study. Finally, 1882 patients with a mean age of 41.3 ± 19.2 years (49.89% male) were included with a definite diagnosis of VVS based on the Calgary Symptom Score who underwent HUTT. The baseline characteristics of patients are summarized in Table 1.

**Table 1:** Baseline characteristics of patients with VVS

	Total patients (N = 1,882)
Age (years) mean ± SD	41.3 ± 19.2
Male sex n (%)	938 (49.8)
Body mass index (kg/m^2^) mean ± SD	25.5 ± 4.9
Lifetime VVS episodes	
1	473 (26.3)
2	374 (20.8)
3+	950 (52.9)
Diabetes mellitus (%)	120 (6.4)
Hypertension (%)	334 (17.7)
Dyslipidemia (%)	324 (17.2)
Valvular heart disease (%)	57 (3.0)
Anemia (%)	305 (16.2)
Chronic kidney disease (%)	39 (2.1)
Arrhythmia (%)	113 (6.0)
Permanent pacemaker (%)	8 (0.4)
Implantable cardioverter-defibrillator (%)	8 (0.4)
History of cardiopulmonary resuscitation (%)	8 (0.4)
History of myocardial infarction (%)	63 (3.3)
Syncope family history	150 (8.0)
Sudden cardiac death family history	103 (5.5)

Data are presented as mean ± standard deviation or number (percentage)

Abbreviations: VVS: vasovagal syncope, SD: standard deviation

### Neurologic assessment

The proportions of using brain MRI, EEG, and brain MRI/EEG were 810/1882 (43.04%, 95% CI 40.82–45.29%), 985/1882 (52.34%, 95% CI 50.08–54.59%), and 1166/1882 (61.96%, 95% CI 59.74–64.18%), respectively. EEG and brain MRI were normal in patients who underwent these neurological assessments. Association between variables and misuse of MRI, EEG, and MRI/EEG are available in Table 2.

**Table 2:** Predictors of neurologic examination in patients with VVS

	Brain MRI		EEG		Brain MRI/EEG	
	Yes (N = 810)	No (N = 1,072)	Yes (N = 985)	No (N = 897)	Yes (N = 1,166)	No (N = 716)
Head trauma	173 (21.36) ^*^	181 (16.88)	212 (21.52) ^**^	142 (15.83)	246 (21.10) ^**^	108 (15.08)
Male sex	349 (43.09) ^***^	589 (55.05)	439 (44.57) ^***^	499 (55.75)	525 (45.02) ^***^	413 (57.84)
First visited by neurologist	194 (23.95) ^***^	88 (8.21)	232 (23.55) ^***^	50 (5.57)	254 (21.78) ^***^	28 (3.91)
Presence of specific triggers	344 (42.47)	427 (39.83)	402 (40.81)	369 (41.14)	477 (40.91)	294 (41.06)
Tongue bite	45 (5.56) ^**^	31 (2.89)	54 (5.48) ^**^	22 (2.45)	58 (4.97) ^*^	18 (2.51)
Gaze disturbance during syncope	81 (10.00) ^**^	66 (6.16)	104 (10.56) ^***^	43 (4.79)	116 (9.95) ^***^	31 (4.33)
Urinary incontinence after syncope	61 (7.53) ^*^	53 (4.94)	85 (8.63) ^***^	29 (3.23)	92 (7.89) ^***^	22 (3.07)
Movements during and after attacks	101 (12.47) ^***^	81 (7.56)	125 (12.69) ^***^	57 (6.35)	136 (11.66) ^***^	46 (6.42)
Chest pain before syncope	83 (10.25) ^**^	149 (13.90)	101 (10.25) ^**^	131 (14.60)	128 (10.98) ^*^	104 (14.52)
Amnesia after syncope	123 (15.18)	137 (12.78)	161 (16.34) ^**^	99 (11.04)	184 (15.78) ^**^	76 (10.61)
Headache after syncope	238 (29.38) ^*^	269 (25.09)	290 (29.44) ^*^	217 (24.19)	338 (28.99) ^*^	169 (23.60)
Autonomic activation before syncope	384 (47.41)	534 (49.81)	481 (48.83)	437 (48.72)	571 (48.97)	347 (48.46)
Movements in HUTT	115 (14.20) ^*^	116 (10.82)	145 (14.72) ^**^	86 (9.59)	169 (14.49) ^***^	62 (8.66)

Data are presented as number (percentage). MRI: magnetic resonance imaging, EEG: electroencephalography, HUTT: head-up tilt table

* *P* < 0.05, ** *P* < 0.01, *** *P* < 0.001

#### Brain MRI

Male sex was significantly lower in patients who underwent brain MRI (43.1% vs. 55.1%, *P* < 0.001). Head trauma (21.4% vs. 16.9%, *P* = 0.015), the first visit by neurologists (23.9% vs. 8.2%, *P* < 0.001), tongue bite (5.6% vs. 2.9%, *P* = 0.004), gaze disturbance during syncope (10.0% vs. 6.2%, *P* = 0.002), urinary incontinence (7.53% vs. 4.94%, *P* = 0.024), headache (29.4% vs. 25.1%, *P* = 0.041), and movements during VVS attacks and HUTT (tonic-clonic movements and myoclonus; 12.47% vs. 7.56% in VVS and 14.20% vs. 10.82% in HUTT) were significantly higher in patients with previous brain MRI compared to ones without MRI. However, chest pain was significantly lower in patients who underwent brain MRI (10.25% vs. 13.90%, *P* = 0.017).

#### EEG

Similar to brain MRI, the male sex was significantly lower in patients who underwent EEG (44.57% vs. 55.75%, *P* < 0.001). Moreover, head trauma (21.52% vs. 15.83%, *P* = 0.002), the first visit by a neurologist (23.55% vs. 5.57%, *P* < 0.001), tongue bite (5.48% vs. 2.45%, *P* = 0.001), gaze disturbance (10.56% vs. 4.79%, *P* < 0.001), urinary incontinence after syncope (8.63% vs. 3.23%, *P* < 0.001), movements during and after attacks (12.69% vs. 6.35%, *P* < 0.001), headache after syncope (29.44% vs. 24.19%, *P* = 0.010), and movements in HUTT (14.72% vs. 9.59%, *P* = 0.001) were significantly higher in patients with previous EEG compared to ones without EEG. However, chest pain was significantly lower in patients who underwent EEG (10.25% vs. 14.60%, *P* = 0.004). Although amnesia was not different between patients with or without previous brain MRI, patients with previous EEG had higher amnesia compared to patients without EEG (16.3% vs. 11.0%, *P* = 0.001).

#### Brain MRI/EEG

The difference in presence of initiating factors and autonomic symptoms between patients with previous brain MRI or EEG and patients without neurologic examinations were not statistically significant (*P* > 0.05; Table 2). Similar to previous comparisons, the male sex contributed more to patients who did not undergo neurologic examination (57.8% vs. 45.0%, *P* < 0.001). Head trauma, referred by neurologists, tongue bite, gaze disturbance, urinary incontinence, movements (VVS and HUTT), amnesia, and headache were higher in patients with previous neurologic examinations and were all statistically significant.

### Association between clinical features and brain MRI or EEG overemployment

**Fig. 1:**
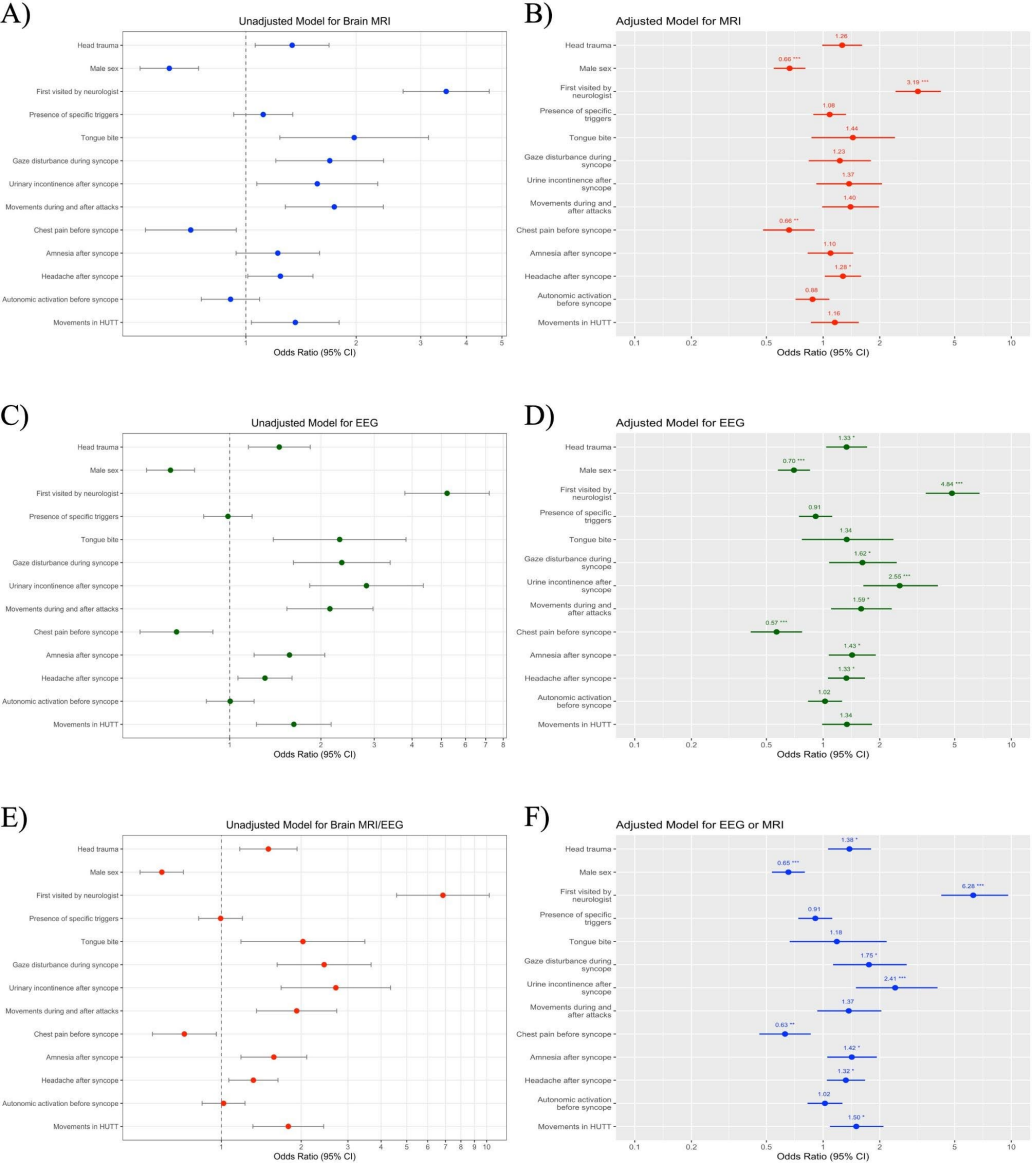
Associations between clinical features of patients with VVS and previous **(A)** brain MRI (unadjusted), **(B)** brain MRI (adjusted), **(C)** EEG (unadjusted), **(D)** EEG (adjusted), **(E)** brain MRI or EEG (unadjusted), and **(F)** brain MRI or EEG (adjusted)

Univariate logistic regression for brain MRI, EEG, and brain MRI/EEG was significant for most of the variables. Table 3 presents all associations. Figure 1 A presents univariate regression for brain MRI in which there was no statistically significant association between brain MRI use and the presence of specific triggers (OR 1.115, 95% CI 0.926 to 1.342, *P* = 0.249), amnesia after syncope (OR 1.222, 95% CI 0.940 to 1.589, *P* = 0.135), and autonomic activation before syncope (OR 0.908, 95% CI 0.756 to 1.090, *P* = 0.301). However, amnesia after syncope was significantly related to EEG in patients with VVS (OR 1.575, 95% CI 1.204 to 2.060, *P* = 0.001, Fig. 1C). The univariate results for brain MRI/EEG were similar to EEG which is illustrated in Fig. 1E.

**Table 3:** Univariate (unadjusted) associations of variables with neurologic evaluation

	Brain MRI		EEG		Brain MRI/EEG	
	OR [95% CI]	*P*	OR [95% CI]	*P*	OR [95% CI]	*P*
Head trauma	1.337 [1.060–1.686]	0.014	1.458 [1.153–1.844]	0.002	1.505 [1.174–1.930]	0.001
Male sex	0.618 [0.514–0.743]	< 0.001	0.638 [0.532–0.766]	< 0.001	0.597 [0.494–0.720]	< 0.001
First visited by neurologist	3.522 [2.685–4.619]	< 0.001	5.219 [3.786–7.194]	< 0.001	6.843 [4.575–10.236]	< 0.001
Presence of specific triggers	1.115 [0.926–1.342]	0.249	0.987 [0.821–1.186]	0.886	0.994 [0.822–1.201]	0.948
Tongue bite	1.975 [1.238–3.151]	0.004	2.307 [1.393–3.820]	0.001	2.030 [1.186–3.474]	0.010
Gaze disturbance during syncope	1.694 [1.207–2.376]	0.002	2.344 [1.623–3.386]	< 0.001	2.441 [1.624–3.670]	< 0.001
Urinary incontinence after syncope	1.566 [1.071–2.290]	0.021	2.827 [1.836–4.353]	< 0.001	2.702 [1.681–4.344]	< 0.001
Movements during and after attacks	1.743 [1.281–2.371]	< 0.001	2.142 [1.544–2.972]	< 0.001	1.923 [1.358–2.724]	< 0.001
Chest pain before syncope	0.707 [0.532–0.941]	0.017	0.668 [0.506–0.881]	0.004	0.726 [0.550–0.958]	0.023
Amnesia after syncope	1.222 [0.940–1.589]	0.135	1.575 [1.204–2.060]	0.001	1.578 [1.186–2.099]	0.002
Headache after syncope	1.242 [1.012–1.524]	0.038	1.308 [1.065–1.605]	0.010	1.321 [1.067–1.636]	0.011
Autonomic activation before syncope	0.908 [0.756–1.090]	0.301	1.004 [0.838–1.204]	0.960	1.020 [0.847–1.229]	0.831
Movements in HUTT	1.364 [1.035–1.797]	0.028	1.628 [1.226–2.162]	0.001	1.788 [1.315–2.431]	< 0.001

Data are presented as odds ratio [95% confidence interval]. MRI: magnetic resonance imaging, EEG: electroencephalography, HUTT: head-up tilt table, OR: odds ratio, CI: confidence interval

Table 4 presents details of associations between variables and brain MRI, EEG, or brain MRI/EEG. The multivariable logistic regression model for brain MRI showed positive associations for MRI with neurologist visit (OR 3.191, 95% CI 2.425 to 4.225, *P* < 0.001) and headache after syncope (OR 1.275, 95% CI 1.021 to 1.592, *P* = 0.032). There were significant negative associations with the male sex (OR 0.665, 95% CI 0.547 to 0.806, *P* < 0.001) and chest pain before syncope (OR 0.660, 95% CI 0.480 to 0.902, *P* = 0.010). Figure 1B illustrates ORs and CIs for associations between all variables and brain MRI.

**Table 4:** Multivariable (adjusted) model for the association of variables with neurologic evaluation

	Brain MRI		EEG		Brain MRI/EEG	
	OR [95% CI]	*P*	OR [95% CI]	*P*	OR [95% CI]	*P*
Head trauma	1.263 [0.989–1.611]	0.061	1.333 [1.038–1.714]	0.025	1.380 [1.063–1.801]	0.016
Male sex	0.665 [0.547–0.806]	< 0.001	0.701 [0.576–0.852]	< 0.001	0.655 [0.535–0.800]	< 0.001
First visited by neurologist	3.191 [2.425–4.225]	< 0.001	4.839 [3.513–6.783]	< 0.001	6.277 [4.244–9.635]	< 0.001
Presence of specific triggers	1.084 [0.887–1.324]	0.430	0.913 [0.745–1.118]	0.38	0.909 [0.739–1.119]	0.369
Tongue bite	1.440 [0.867–2.408]	0.160	1.335 [0.771–2.365]	0.309	1.183 [0.665–2.179]	0.576
Gaze disturbance during syncope	1.228 [0.839–1.796]	0.290	1.619 [1.077–2.461]	0.022	1.753 [1.130–2.783]	0.014
Urinary incontinence after syncope	1.374 [0.920–2.055]	0.120	2.551 [1.636–4.074]	< 0.001	2.415 [1.494–4.055]	0.001
Movements during and after attacks	1.399 [0.989–1.981]	0.058	1.593 [1.103–2.317]	0.014	1.369 [0.931–2.038]	0.115
Chest pain before syncope	0.660 [0.480–0.902]	0.010	0.566 [0.413–0.774]	< 0.001	0.628 [0.459–0.860]	0.004
Amnesia after syncope	1.095 [0.828–1.447]	0.522	1.427 [1.071–1.905]	0.015	1.421 [1.053–1.930]	0.023
Headache after syncope	1.275 [1.021–1.592]	0.032	1.330 [1.061–1.669]	0.013	1.321 [1.046–1.672]	0.020
Autonomic activation before syncope	0.879 [0.715–1.080]	0.220	1.024 [0.831–1.261]	0.825	1.023 [0.826–1.266]	0.836
Movements in HUTT	1.155 [0.861–1.548]	0.334	1.338 [0.988–1.820]	0.061	1.501 [1.087–2.093]	0.015

Data are presented as odds ratio [95% confidence interval]. MRI: magnetic resonance imaging, EEG: electroencephalography, HUTT: head-up tilt table, OR: odds ratio, CI: confidence interval

**Fig. 2:**
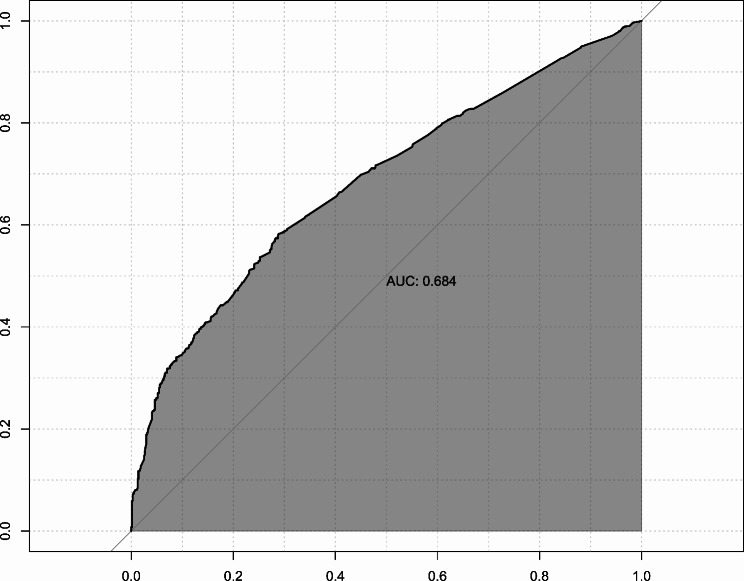
Receiver operating characteristic curve discriminating patients with previous brain MRI or EEG from those without previous brain MRI or EEG

Regarding EEG, in addition to neurologist visits, headache after syncope, male sex, and chest pain, there were significant correlations between EEG and head trauma (OR 1.333, 95% CI 1.038 to 1.714, *P* = 0.025), gaze disturbance during syncope (OR 1.619, 95% CI 1.077 to 2.461, *P* = 0.022), urinary incontinence after syncope (OR 2.551, 95% CI 1.636 to 4.074, *P* < 0.001), movements during and after syncope (OR 1.593, 95% CI 1.103 to 2.317, *P* = 0.014), and amnesia after syncope (OR 1.427, 95% CI 1.071 to 1.905, *P* = 0.015). Figure 1D illustrates ORs and CIs for associations between all variables and EEG. Finally, the combined MRI/EEG model analysis resulted in the same significant associations, details of which are available in Table 4; Fig. 1F. The model that used brain MRI/EEG as a dependent variable showed acceptable discrimination power with an AUC of 0.684 [95% CI 0.660–0.708] in discriminating patients with previous brain MRI/EEG from those without previous brain MRI/EEG (Fig. 2).

## Discussion

In this study, we evaluated misuse of neurologic assessments (brain MRI or EEG) in a tertiary syncope unit in patients with VVS. We found high rates of overuse in MRI, EEG, and MRI/EEG. First visit by neurologists, head trauma, female sex, tongue bite, gaze disturbance, urinary incontinence, tonic or clonic movements, myoclonus, amnesia, and headache were higher in patients with MRI or EEG while there was no difference in the presence of initiating factors and autonomic symptoms. Multivariate analysis also showed that head trauma, previous neurologist visit, gaze disturbance during syncope, urinary incontinence, amnesia, post-syncope headache, and movements in HUTT have all positive correlations with performing either brain MRI or EEG. On the other hand, male sex and chest pain before syncope had significant negative associations with them.

Neurologic examination is not recommended as a routine diagnostic method in patients with syncope. The ESC guidelines published in 2018 recommend neurological evaluations in patients whose syncope is due to an autonomic disorder or in case of high epilepsy suspicion [3]. However, brain MRI is recommended only in patients whose neurological examination shows evidence of parkinsonism, ataxia, or cognitive impairment (class of recommendation [CoR]: 1). In patients with syncope without suspicion of neurological causes, EEG, carotid ultrasound, and brain MRI are not indicated (CoR: 3). Similarly, the American guidelines published in 2017 does not recommend brain MRI in the routine evaluation of syncope patients [2]. It can be considered as a neurological evaluation only in patients who have focal neurological deficits. The use of EEG for recognition of abnormal patterns during HUTT has been introduced [12, 13], however, there were several limitations to these studies, hence limiting its clinical application for the diagnosis of VVS. For instance, the study by van Dijk [13] lacked a control group, and the patients were highly selected and in a highly monitored situation that may not be similar to spontaneous VVS or syncope from other causes. This leads to questions usefulness of EEG evaluation in VVS and its real-world utility [14]. Therefore, similar to brain MRI, nowadays, EEG does not play a role in the normal routine examination of patients with syncope. While the main diagnostic use of EEG is for seizures, it should be noted that a negative EEG could not rule out seizures. This is mainly due to the fact that interictal discharges (IEDs) could not be recorded and also, focal epilepsy IEDs are not measurable by standard EEGs. Overall, it is important to emphasize that clinical presentation plays the most important role in the diagnosis of a seizure while EEG is used in routine diagnostic workups, repeating of which could increase the sensitivity for seizure diagnosis [15].

A retrospective study conducted by Choi et al. [16] assessed the effectiveness of several diagnostic tests in 160 children with TLOC due to different reasons. In this study, it was shown that while EEG had 80% sensitivity and 80% specificity for the diagnosis of epileptic seizures, these were only 16.7% and 36.4% for VVS, respectively. Many other studies have also investigated the misuse of neurologic assessments in patients with syncope; however, nearly none of them have been specific to patients with VVS. In a study conducted by Kapoor et al. in 1983 [17], EEG was performed for 101 out of 204 patients with syncope but it was diagnostic in only one case, highlighting the ineffectiveness of EEG in patients with syncope. Mendu et al. [18] found that EEG and MRI helped diagnosis of syncope in two and three cases out of 1920 patients diagnosed with syncope, respectively. In a study by Johnson et al. [19], among 1038 patients with symptoms similar to syncope, a definitive diagnosis of syncope was given to 167 patients. Brain MRI was not diagnostic in any patient with syncope. In a study by Abubakr et al. [20], among patients with syncope, only 1.46% of EEGs showed epileptiform discharges, which was the same percentage as its occurrence in the healthy population. Hence, it was shown that EEG does not have any added value for VVS diagnosis other than its use in the diagnosis of seizures. A retrospective study [21] of patients with suspected syncope, found that none of the EEGs suggested epileptiform activity. In a retrospective study by Pires et al. [22] evaluating hospitalized patients with syncope EEG was useful in diagnosis in only 6 patients (2%) whose history was not consistent with syncope. While blood pressure measurement was helpful in 30% of cases.

Suspicion of seizures is the most important underlying reason to perform MRI and EEG in patients presenting with TLOC. We found a significantly higher rate of brain MRI and EEG in patients referred by neurologists, emphasizing suspicion of seizure in VVS patients. In a study by Sheldon et al. [23], historical data showed promising results in differentiating causes of TLOC including seizures and syncope. Relying only on symptoms resulted in 94% specificity and 94% sensitivity in diagnosing seizures. Symptoms similar to seizures have been reported in patients with VVS. In a retrospective study, they detected seizure-like activities in the HUTT-induced VVS [24]. However, it was not correlated with hemodynamic changes during HUTT and the severity of VVS episodes. In another study, Passman et al. [25] reviewed HUTT results in patients with syncope. Neurological events were present in 8% of patients including tonic-clonic seizure-like activity, focal seizures, dysarthria, aphasia, unilateral extremity dysesthesia, and reproduction of temporal lobe epilepsy symptoms. All in all, although some neurological activities are present in patients with VVS, precise history-taking can differentiate VVS from seizures.

Performing brain MRI and EEG may have some drawbacks for patients with VVS as well. First, the cost of neurological evaluations, especially MRI, is very high in many countries and imposes a burden on both patients and the healthcare systems. The financial burden of inappropriate brain MRI in Iran in 2017 was assessed in a cross-sectional study and it was shown that 21% of brain MRIs are without indication, leading to about 100,000$ annual cost. This emphasizes avoiding the use of MRI without indication and also highlights the need for cost-effectiveness analysis of MRI in the evaluation of syncope. Additionally, although VVS is not an emergency condition, the use of neurologic evaluations might delay the diagnosis and subsequently the treatment of VVS, including lifestyle changes and pharmacologic treatments [2, 3, 26].

Neurologic findings are prevalent in patients with VVS. According to recent guidelines, however, neurologic assessment is only indicated in cases of high epilepsy suspicion. We found a high rate of tonic or clonic movements, myoclonus, headache, and amnesia in the typical VVS population. Thus, physicians who visit syncope patients for the first time should use screening tools for this disease such as CSSS or HUTT to prevent the inappropriate use of neurological evaluations [11]. Patients with susceptibility to VVS should be screened using these tools. CSSS showed 91% specificity and 89% sensitivity in diagnosing VVS in its first evaluation [11], making it a non-invasive and easy-to-use tool for VVS screening, especially in the younger population [27].

## Limitations

Although this study is the first to investigate the misuse of neurological assessments in patients with VVS and with an acceptable sample size, it also has some limitations. For the entire population of this study, HUTT was performed, while HUTT is not required for the diagnosis of VVS, and this causes people who have a definite diagnosis of VVS but HUTT was not performed for them, to be missed. Moreover, this is a single-center study and the results may not be generalized to all populations.

## Conclusions

While about half of the patients diagnosed with VVS underwent EEG or MRI, the factors associated with this were head trauma, referring by the neurologist, headaches, and typical seizure presentations such as tongue bite, urinary incontinence, and tonic-clonic movements. Clinicians should follow the guidelines and avoid administrating brain MRI or EEG in case of history and physical examination consistent with VVS, as they have no place in the routine evaluation of these patients.

## Data Availability

Data generated and/or analyzed in this study are available at reasonable request from the corresponding author.

## References

[1] Chen LY, Shen WK, Mahoney DW, Jacobsen SJ, Rodeheffer RJ (2006). Prevalence of syncope in a population aged more than 45 years. Am J Med.

[2] Shen WK, Sheldon RS, Benditt DG, Cohen MI, Forman DE, Goldberger ZD (2017). 2017 ACC/AHA/HRS Guideline for the evaluation and management of patients with Syncope: executive summary: a report of the American College of Cardiology/American Heart Association Task Force on Clinical Practice guidelines and the Heart Rhythm Society. J Am Coll Cardiol.

[3] Brignole M, Moya A, de Lange FJ, Deharo JC, Elliott PM, Fanciulli A (2018). 2018 ESC guidelines for the diagnosis and management of syncope. Eur Heart J.

[4] Kenny RA, Ingram A, Bayliss J, Sutton R (1986). Head-up tilt: a useful test for investigating unexplained syncope. Lancet.

[5] Parry SW, Gray JC, Newton JL, Reeve P, O’Shea D, Kenny RA (2008). Front-loaded’ head-up tilt table testing: validation of a rapid first line nitrate-provoked tilt protocol for the diagnosis of vasovagal syncope. Age Ageing.

[6] Brignole M, Menozzi C, Bartoletti A, Giada F, Lagi A, Ungar A (2006). A new management of syncope: prospective systematic guideline-based evaluation of patients referred urgently to general hospitals. Eur Heart J.

[7] Sarasin FP, Junod AF, Carballo D, Slama S, Unger PF, Louis-Simonet M (2002). Role of echocardiography in the evaluation of syncope: a prospective study. Heart.

[8] Sakaguchi S, Shultz JJ, Remole SC, Adler SW, Lurie KG, Benditt DG (1995). Syncope associated with exercise, a manifestation of neurally mediated syncope. Am J Cardiol.

[9] Sadeghian S, Aminorroaya A, Tajdini M (2021). The Syncope Unit of Tehran Heart Center. Eur Heart J.

[10] Poorhosseini H, Abbasi SH (2018). The Tehran Heart Center. Eur Heart J.

[11] Sheldon R, Rose S, Connolly S, Ritchie D, Koshman ML, Frenneaux M (2006). Diagnostic criteria for vasovagal syncope based on a quantitative history. Eur Heart J.

[12] Ammirati F, Colivicchi F, Di Battista G, Garelli FF, Santini M (1998). Electroencephalographic correlates of vasovagal syncope induced by head-up tilt testing. Stroke.

[13] van Dijk JG, Thijs RD, van Zwet E, Tannemaat MR, van Niekerk J, Benditt DG (2014). The semiology of tilt-induced reflex syncope in relation to electroencephalographic changes. Brain.

[14] Solbiati M, Sheldon R (2014). Syncope: how the EEG helps in understanding clinical findings. Brain.

[15] Brigo F (2011). An evidence-based approach to proper diagnostic use of the electroencephalogram for suspected seizures. Epilepsy Behav.

[16] Choi YJ, Han MY, Lee EH (2020). Children with transient loss of consciousness: clinical characteristics and the effectiveness of diagnostic tests. Pediatr Neonatol.

[17] Kapoor WN, Karpf M, Wieand S, Peterson JR, Levey GS (1983). A prospective evaluation and follow-up of patients with syncope. N Engl J Med.

[18] Mendu ML, McAvay G, Lampert R, Stoehr J, Tinetti ME (2009). Yield of diagnostic tests in evaluating syncopal episodes in older patients. Arch Intern Med.

[19] Johnson PC, Ammar H, Zohdy W, Fouda R, Govindu R (2014). Yield of diagnostic tests and its impact on cost in adult patients with syncope presenting to a community hospital. South Med J.

[20] Abubakr A, Wambacq I (2005). The diagnostic value of EEGs in patients with syncope. Epilepsy Behav.

[21] Poliquin-Lasnier L, Moore FG (2009). EEG in suspected syncope: do EEGs ordered by neurologists give a higher yield?. Can J Neurol Sci.

[22] Pires LA, Ganji JR, Jarandila R, Steele R (2001). Diagnostic patterns and temporal trends in the evaluation of adult patients hospitalized with Syncope. Arch Intern Med.

[23] Sheldon R, Rose S, Ritchie D, Connolly SJ, Koshman M-L, Lee MA (2002). Historical criteria that distinguish syncope from seizures. J Am Coll Cardiol.

[24] Song PS, Kim JS, Park J, Yim HR, Huh J, Kim JH (2010). Seizure-like activities during head-up tilt test-induced syncope. Yonsei Med J.

[25] Passman R, Horvath G, Thomas J, Kruse J, Shah A, Goldberger J (2003). Clinical spectrum and prevalence of neurologic events provoked by tilt table testing. Arch Intern Med.

[26] Behnoush AH, Yazdani K, Khalaji A, Tavolinejad H, Aminorroaya A, Jalali A et al. Pharmacologic prevention of recurrent vasovagal syncope: a systematic review and network meta-analysis of randomized controlled trials. Heart Rhythm. 2022. 10.1016/j.hrthm.2022.12.010 36509319

[27] Expósito V, Guzmán JC, Orava M, Armaganijan L, Morillo CA (2013). Usefulness of the Calgary Syncope Symptom Score for the diagnosis of vasovagal syncope in the elderly. EP Europace.

